# Strategies for Improving Polio Surveillance Performance in the Security-Challenged Nigerian States of Adamawa, Borno, and Yobe During 2009–2014

**DOI:** 10.1093/infdis/jiv530

**Published:** 2016-04-02

**Authors:** Abdullahi Walla Hamisu, Ticha Muluh Johnson, Kehinde Craig, Pascal Mkanda, Richard Banda, Sisay G. Tegegne, Ajiboye Oyetunji, Nuhu Ningi, Said M. Mohammed, Mohammed Isa Adamu, Khalid Abdulrahim, Peter Nsubuga, Rui G. Vaz, Ado J. G. Muhammed

**Affiliations:** 1 World Health Organization, Country Representative Office; 2 National Primary Health Care Development Agency, Abuja, Nigeria; 3 World Health Organization, Regional Office for Africa, Brazzaville, Congo; 4 Global Public Health Solutions, Atlanta, Georgia

**Keywords:** security challenge, cross border importations, community informants, surveillance performance

## Abstract

***Background.*** The security-challenged states of Adamawa, Borno, and Yobe bear most of the brunt of the Boko Haram insurgency in Nigeria. The security challenge has led to the killing of health workers, destruction of health facilities, and displacement of huge populations. To identify areas of polio transmission and promptly detect possible cases of importation in these states, polio surveillance must be very sensitive.

***Methods.*** We conducted a retrospective review of acute flaccid paralysis surveillance in the security-compromised states between 2009 and 2014, using the acute flaccid paralysis database at the World Health Organization Nigeria Country Office. We also reviewed the reports of surveillance activities conducted in these security-challenged states, to identify strategies that were implemented to improve polio surveillance.

***Results.*** Environmental surveillance was implemented in Borno in 2013 and in Yobe in 2014. All disease surveillance and notification officers in the 3 security-challenged states now receive annual training, and the number of community informants in these states has dramatically increased. Media-based messaging (via radio and television) is now used to sensitize the public to the importance of surveillance, and contact samples have been regularly collected in both states since 2014.

***Conclusions.*** The strategies implemented in the security-challenged states improved the quality of polio surveillance during the review period.

Nigeria has recorded tremendous progress in polio eradication. The number of polio cases in 2014 was 6, compared with 53 in 2013. At the time of writing, wild poliovirus (WPV) type 3 was last isolated in Nigeria during November 2012. In addition, the number of genetic clusters of WPV decreased from 8 in 2012 to just 1 in 2014 [1].

In Nigeria, the quality of acute flaccid paralysis (AFP) surveillance, as gauged by the non–polio-associated AFP rate and the percentage of AFP cases with adequate stool specimen collection, has been impressive for the past 10 years. In 2013, for instance, the country recorded a non–polio-associated AFP rate of 12.1 cases per 100 000 children <15 years old (target, 2.0 cases per 100 000 children <15 years old), and 96.9% of AFP cases had 2 stool samples that were collected within 14 days of paralysis onset and reached the laboratory in good condition (target, 80% of cases) [2]. Despite this performance, surveillance gaps at subnational levels, such as inadequate active case searching, poor documentation, and inadequate clinician and community sensitization, still remain [3, 4].

The northeast geopolitical zone of Nigeria comprises 6 states: Adamawa, Bauchi, Borno, Gombe, Taraba, and Yobe. This zone has an estimated population of 25 million [5]. Adamawa, Borno, and Yobe bear most of the brunt of challenges to security as a result of the Boko Haram insurgency. The compromised security has resulted in the killing of health workers, the destruction of health facilities, and the displacement of large numbers of individuals, all of which have negatively affected polio eradication activities, including surveillance. The security challenges have been sufficiently intense, widespread, and persistent to warrant the declaration of state of emergency in these states [6, 7]. These states are also at high risk for polio transmission, because even in the absence of security challenges vaccine noncompliance was rampant in these areas, leading to widespread outbreaks of WPV and vaccine-derived poliovirus (VDPV) infection. In January 2012, a report from the Global Polio Independent Monitoring Board delineated 4 distinct poliovirus sanctuaries in Nigeria, which included the Borno-Yobe axis and the Kano-Sokoto axis [8, 9].

To identify areas of polio transmission in Adamawa, Borno, and Yobe, polio surveillance must be very sensitive. These states also have international borders with Cameroon, Chad, and Niger, which are also affected by the insurgency. The possibility of cross-border importations further makes it paramount that polio surveillance be enhanced, especially considering that Cameroun reported 4 cases of WPV infection in 2013 and 5 cases in 2014, with the date of onset of the most recent case being 9 July 2014 [10]. Here, we describe how the implementation of key strategies in Adamawa, Borno, and Yobe influenced the quality of polio surveillance.

## METHODS

### Strategies to Improve Polio Surveillance

#### Environmental Surveillance

Environmental surveillance involves testing sewage samples for the presence of poliovirus. Environmental surveillance in Borno began in October 2013, with 3 collection sites initially: 2 in the municipal council area and 1 in the Jere local government area (LGA). Both the Jere LGA and the municipal council area are metropolitan and are categorized as having a very high risk for polio transmission. Every collection site has 2 trained sample collectors (a main collector and a backup or assistant), and sample collection was done once monthly at every site. One additional collection site was later added during a review of the sites in November 2013. In Yobe, environmental surveillance started in November 2014 with 2 collection sites in the state capital, Damaturu LGA, and 1 site in Bade LGA. Environmental surveillance had not been established in Adamawa as of December 2014. All environmental samples in Nigeria are transported according to World Health Organization (WHO) guidelines for sample transport [11] to Ibadan National Polio Laboratory for analysis.

#### Training of Disease Surveillance and Notification Officers (DSNOs)

All DSNOs in the 3 states were trained on integrated disease surveillance and response annually. This was in addition to on the job training during supervision and also during the monthly DSNO review meetings. The formal training was conducted by the state teams, comprising state ministry of health officials and other partners, such as the WHO.

#### Engagement and Sensitization of Community Informants on Polio Surveillance

Informants are people who own or manage places in the nonformal health sector where parents with AFP may most likely seek assistance or consult. Examples are patent medicine vendors, traditional and spiritual healers, traditional bonesetters, and traditional birth attendants. All 3 states were able to engage and sensitize more community informants on surveillance. The state team engaged informants after meetings with different associations of nonformal healthcare providers, such as the patent medicine vendor association. The sensitization emphasized the AFP case definition and the roles informants could play in AFP case identification and reporting. At the end of sensitization, AFP picture posters and case definitions were distributed to all informants.

#### Sensitization of the Public on Polio Surveillance Through the Media

Sensitization of the public through radio broadcasts, phone-in programs, and jingles were aired at prime times. The discussions and jingles broadcast on radio and television focused on importance of immunization, the description of AFP cases, and the need to report. Myths and fears about immunization were allayed during phone-in programs. In Adamawa, the television station used was Gotel, while in Borno and Yobe, the radio stations used were Borno radio and the Yobe Broadcasting Corporation, respectively.

#### Engagement and Sensitization of Community-Based Organizations (CBOs)

Select CBOs in these states were also sensitized on polio surveillance. Selection of the CBOs was mainly based on their influence in the communities. For instance, the Federation of Muslim Women Association of Nigeria was selected because it is a popular and influential group in these states. The association has an influence on mothers through its preaching platforms, as well as its house-to-house sensitization activities. The association of commercial motorcycle riders is another influential group whose membership is extensive, reaching even the most-remote areas. Sensitization of these groups was focused on their role in supporting polio eradication initiative activities including surveillance.

#### Collection of Stool Specimens From Contacts

Collection of stool specimens from 3 contacts of every individual with AFP was initiated in Borno and Yobe in 2014 with the aim of further improving the sensitivity of surveillance.

#### Data Collection and Analysis

A retrospective review of the quality of AFP surveillance in Adamawa, Borno, and Yobe was conducted between 2009 and 2014. The 2 core AFP surveillance performance indicators—the non–polio-associated AFP rate and the percentage of AFP cases with adequate stool specimen collection—were analyzed using the AFP database at the WHO Country Office, Nigeria. We also reviewed the reports of surveillance activities conducted in the security-challenged states of Adamawa, Borno, and Yobe, to identify strategies that were implemented to improve polio surveillance. Challenges to surveillance were then identified.

## RESULTS

**Figure 1.**
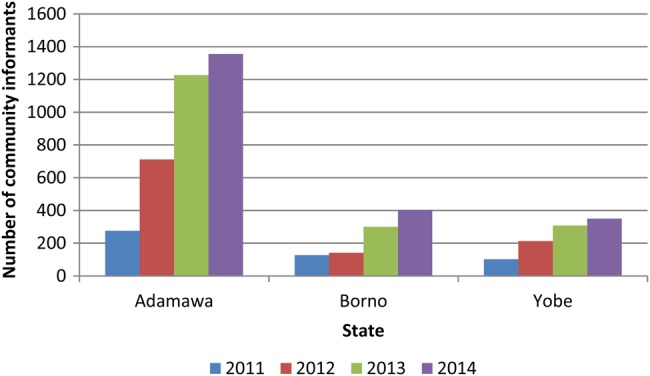
Distribution of community informants in the security-compromised Nigerian states of Adamawa, Borno, and Yobe, 2011–2014.

The number of community informants in the 3 security-challenged states has increased since 2011 (Figure 1). Adamawa consistently expanded its network of community informants at a much higher scale than Borno and Yobe. In all 3 states, the number of community informants was highest in 2014.

The proportion of DSNOs trained in all the security-challenged states in 2013 was <90%. In 2014, however, the proportion rose to 100% in all security-challenged states.

There was detection of WPV type 1 in Borno during 2013. In addition, 9 VDPV isolates were detected in 2013, and 13 were detected in 2014. The number of nonpoliovirus enteroviruses and Sabin virus isolates (ie, poliovirus from the oral polio vaccine) was 18 and 23, respectively. One of the VDPVs from the environmental surveillance in Borno during 2014 was an orphan (ie, there was a ≥1.5% difference in genetic identity to its closest match, indicating a surveillance gap). In Yobe, 1 VDPV was isolated in week 48 of 2014. There was 1 nonpoliovirus enterovirus isolate and 3 Sabin virus isolates in Yobe in 2014. There was no isolation of WPV in Yobe during 2014.

All 3 security-challenged states conducted sensitization of CBOs and airing of jingles. Only Adamawa used television broadcasts to sensitize the public. Borno and Yobe used radio broadcasts.

The main sources of AFP reporting in 2014 in the security-challenged states were health workers, focal persons, community informants, and vaccination teams. More AFP cases were reported by community informants in Adamawa than in the other 2 states. AFP reporting by community informants in Adamawa and Borno was higher than the average for the country.

**Figure 2.**
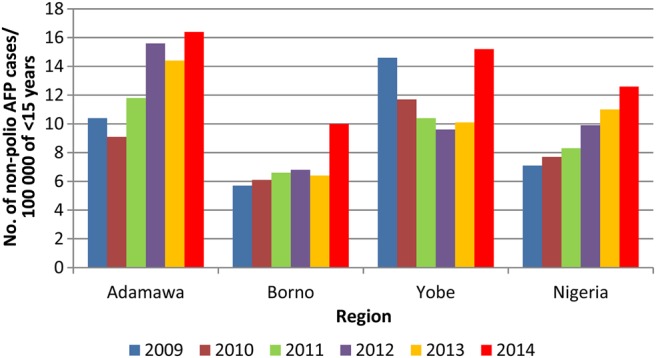
Trend of non–polio-associated acute flaccid paralysis (AFP) rates in the security-challenged Nigerian states of Adamawa, Borno, and Yobe, 2009–2014.

The minimum requirement of AFP surveillance sensitivity, as measured by the non–polio-associated AFP rate (≥2.0 cases per 100 000 children aged <15 years), was met in all the security-challenged states during 2009–2014 (Figure 2). The highest level of AFP surveillance sensitivity during the study period in all 3 states occurred during 2014. Of the 3 states, Adamawa had the highest level of AFP surveillance sensitivity during the study period.

Of the 264 AFP cases reported by Borno and Yobe in 2014, 792 contact samples were expected to be collected; of these, 46% were collected and sent to the laboratory for analysis (Table 1).

**Table 1.** Contact Sample Collection in the Security-Challenged Nigerian States of Borno and Yobe During 2014

State	AFP Cases, No.	Contact Sample Collection	
		Expected No.	Percentage of Expected No.
Borno	88	264	43
Yobe	176	528	49
Overall	264	792	46

Abbreviation: AFP, acute flaccid paralysis.

## DISCUSSION

We found that the quality of polio surveillance in the security-challenged states of Adamawa, Borno, and Yobe consistently improved from 2009 to 2014, with the highest quality observed during 2014 in all 3 states. We believe that this high quality is attributable to the implementation of key surveillance activities, including expansion of the reporting network of community informants, training of DSNOs, engagement and sensitization of CBOs, sensitization of the public via the media, establishment of environmental surveillance, and initiation of contact sample collection.

We also found that traditional strategies of improving polio surveillance can be effective even in times of conflict. Such measures as community involvement have sustained a high level of surveillance in other countries affected by conflict, such as Afghanistan and Pakistan, and have even led to interruption of polio transmission during active conflicts in Cambodia, Peru, and Columbia [12]. However, intervention measures to enhance polio surveillance in security-challenged areas need to be country specific if success is to be assured. For instance, while some countries used negotiation of cease-fire agreements, truces, and conflict-free days, others, such as Nigeria, have concentrated on such measures as community engagement and training of surveillance personnel, as rapid assessment of surveillance in other high-risk states consistently found knowledge gaps among surveillance personnel, especially those at the LGA and health facility levels, to be one of the key challenges to quality surveillance [13, 14].

Contact specimen collection was poor owing to population displacement, as well as the compromised security situation.

One of the key public health implications of our findings is that compromised security by itself is not an absolute barrier to achieving quality surveillance. We found that political commitment, adequate resources, and determined and persistent efforts are required for optimal surveillance in the face of security challenges [15].

One of the key limitations of this study is the inadequate documentation of all surveillance activities conducted before and after the onset of insurgency in the affected states. Thus, the contribution of some activities toward improving polio surveillance may be inadvertently overlooked. Likewise, comparison of the polio surveillance situation before and after the insurgency in these states is inhibited as a result of this dearth of information.

We recommend that continuous community and clinician sensitization be enhanced. Documentation of all surveillance activities and archiving of information should be given priority, along with active case searching and timely provision of feedback to all stakeholders. Finally, surveillance field staff, including the DSNOs, field volunteers, and environmental surveillance sample collectors, should receive commendation and encouragement for working in this extremely challenging security situation.
